# PfaSTer: a machine learning-powered serotype caller for *Streptococcus pneumoniae* genomes

**DOI:** 10.1099/mgen.0.001033

**Published:** 2023-06-06

**Authors:** Jonathan T. Lee, Xingpeng Li, Craig Hyde, Paul A. Liberator, Li Hao

**Affiliations:** ^1^​ Vaccine Research & Development, Pfizer Inc., 401 N. Middletown Rd, Pearl River, NY 10965, USA; ^2^​ Early Clinical Development, Pfizer Inc., 1 Portland St, Cambridge, MA 02139, USA

**Keywords:** serotype, machine learning, *Streptococcus pneumoniae*

## Abstract

*
Streptococcus pneumoniae* (pneumococcus) is a leading cause of morbidity and mortality worldwide. Although multi-valent pneumococcal vaccines have curbed the incidence of disease, their introduction has resulted in shifted serotype distributions that must be monitored. Whole genome sequence (WGS) data provide a powerful surveillance tool for tracking isolate serotypes, which can be determined from nucleotide sequence of the capsular polysaccharide biosynthetic operon (*cps*). Although software exists to predict serotypes from WGS data, most are constrained by requiring high-coverage next-generation sequencing reads. This can present a challenge in respect of accessibility and data sharing. Here we present PfaSTer, a machine learning-based method to identify 65 prevalent serotypes from assembled *S. pneumoniae* genome sequences. PfaSTer combines dimensionality reduction from k-mer analysis with a Random Forest classifier for rapid serotype prediction. By leveraging the model’s built-in statistical framework, PfaSTer determines confidence in its predictions without the need for coverage-based assessments. We then demonstrate the robustness of this method, returning >97 % concordance when compared to biochemical results and other *in silico* serotyping tools. PfaSTer is open source and available at: https://github.com/pfizer-opensource/pfaster.

## Data Summary

Training data for PfaSTer were obtained from the Wellcome Sanger Institute Pathogenwatch platform (pathogen.watch). Accession numbers and isolate names are included in Table S1. Genomic data for isolates used in validation were obtained from the NCBI sequence read archive, project PRJEB14267. Sample accessions for these data can be found in Table S2. Code for PfaSTer is available on GitHub at https://github.com/pfizer-opensource/pfaster.

**Impact Statement**

Here we describe the development of PfaSTer, a computational tool for rapid *Streptococcus pneumoniae* serotype prediction that combines k-mer analysis and machine learning. PfaSTer scans a genome sequence for k-mers associated with 65 serotypes, which are utilized by a Random Forest classifier for prediction. While most *in silico* serotype callers rely on high-coverage next-generation sequencing data, we demonstrate that predictions made by PfaSTer are comparable to other published tools while utilizing much less data in the form of assembled genomes. We also show the advantage of PfaSTer’s machine learning component, which leverages the model’s statistical properties to resolve closely related serotypes that other methods cannot. Overall, PfaSTer provides a new means for high-resolution pneumococcal serotype identification, which can have important impacts on shaping future vaccine development.

## Introduction

*Streptococcus pneumoniae* (pneumococcus) presents a major concern to public health, being a common cause of lower respiratory tract infections and pneumonia [1, 2]. Pneumococcal disease is a particular threat to the elderly, largely due to a high mortality risk when contracting pneumonia [1, 3]. Pneumococcal conjugate vaccines (PCVs) can be used to prevent disease [4, 5] by affording protection against common circulating serotypes. In *S. pneumoniae,* serotype is defined by the structure of a capsular polysaccharide and the genes that direct biosynthesis of the polysaccharide encoded at the capsular polysaccharide synthesis (*cps*) operon [6]. To date, at least 100 pneumococcal serotypes carrying unique *cps* sequences have been identified [7], with a fraction of these found to be prevalent in global populations [8]. As the capsular polysaccharide serves as the target of PCVs [4], surveillance of emerging strains through serotyping is important for monitoring efficacy against circulating strains and the development of new multi-valent vaccines [9].

Traditionally, pneumococcal serotyping is performed using serotype-specific monoclonal antibody reagents, either through the Quellung reaction or latex agglutination [10]. While held in high regard, such methods are expensive and laborious. Antibody tests are also often unable to differentiate closely related serotypes [10, 11], and visual assessments of agglutination results are susceptible to subjective interpretation. Furthermore, the need for cell cultures presents a physical barrier for replicating results between research groups. As an alternative, automated pipelines for predicting serotypes from next-generation sequencing (NGS) data have been developed. Since 2016, bioinformatics tools such as PneumoCaT, SeroBA and, more recently, SeroCall and PneumoKITy, have been utilized to effectively identify serotypes *in silico* [12–15]. While their underlying algorithms differ, these methods primarily utilize the same input: raw NGS data from the *cps* locus and a reference *cps* database for different serotypes. By leveraging an abundance of NGS reads, these applications provide robust predictions of the *cps* sequence and, therefore, the *in silico* serotype.

While a powerful resource, high-coverage NGS data can be unwieldy and computationally intensive to work with. Furthermore, such data are not always readily available to researchers. For instance, the PubMLST [16] microbial database contains, to date, >30 000 pneumococcal genomes from submissions around the globe. Many of these assembled genomes lack accompanying NGS data sources and would be incompatible with most of the previously described serotyping tools. To date, only PneumoKITy can take assembled genomes as input, but it has limited capability to distinguish closely related serotypes [15].

**Fig. 1.**
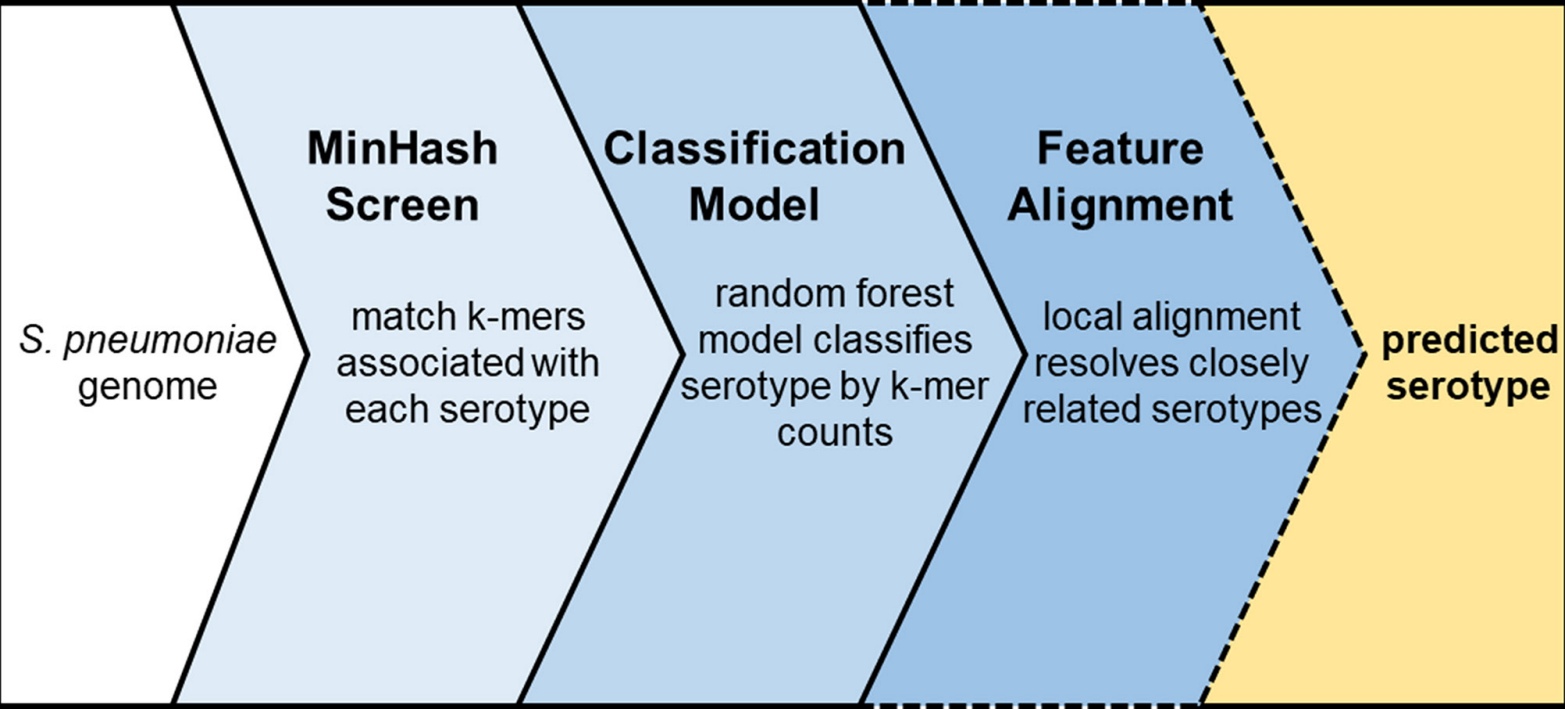
PfaSTer workflow. PfaSTer takes an aligned or assembled *S. pneumoniae* genome sequence (FASTA format) as input. A MinHash (Mash) Screen for k-mers associated with each reference serotype is first performed. The number of k-mers matched to each reference is then passed to a Random Forest classifier to assign a predicted serotype. In cases where the model is unable to discern closely related types, alignment is performed to identify serotype-defining features.

We developed the pneumococcal FASTA serotyper (PfaSTer) to perform high-resolution *in silico* serotyping when constrained to working with assembled or aligned genome sequences. PfaSTer identifies k-mers at the *cps* locus associated with 65 serotypes, which are utilized in machine learning for prediction (Fig. 1). Using a validated dataset of >2000 pneumococcal isolates, we show that PfaSTer is both a fast and highly accurate serotype caller, with predictions comparable to both serological results and other computational methods.

## Methods

### Data sources

Training data for PfaSTer were obtained from the Wellcome Sanger Institute Pathogenwatch platform (https://pathogen.watch) in the form of 3681 *de novo* assembled genomes spanning 65 different serotypes (Table S1, available in the online version of this article). For validation, NGS data for 2065 isolates were obtained from the NCBI sequence read archive (project PRJEB14267). Accessions for these data can be found in Table S2.

### Mash sketch creation

Reference *cps* sequences (previously published and utilized by PneumoCAT [12] and seroBA [13]) were used to develop MinHash sketches [17] of 65 serotypes (Table S3). A sliding window (k-mer) of 15, 30, 60, 70, 80 or 90 nucleotides was used to scan each *cps* sequence, with each k-mer converted to a 128-bit integer using MurmurHash3 (v3.0.0). To account for bidirectionality, both the forward and reverse complemented k-mer were considered and the lexicographically smaller sequence was used for hashing. The k-mers corresponding to the 1000 smallest integer values for each serotype were saved to the sketches.

### Model training and probability thresholding

A Mash Screen [18] was performed for 3681 pneumococcal genomes using the previously described sketches. Each k-mer hash in a sliding window across the genome sequence was compared to those in the reference sketch, and matching k-mers were recorded. The total number of k-mers matched for each serotype, and each k-mer size, were then saved and used as features to train a Random Forest classifier using the R tidymodels package (v0.1.2). To account for class imbalance due to differences in serotype prevalence, overrepresented serotypes were down-sampled to no more than 200 cases for training. Initial model performance for each k-mer size was measured using a 5-fold grid search and computing the average accuracy across 200 Monte Carlo cross-validations for each k-mer size’s optimal hyperparameters (Fig. S1A, B). The model with the best performing k-mer size was then ported to python using the sklearn package (v1.1.1). Hyperparameter tuning was again performed using the sklearn GridSearchCV module (*cv*=3), with optimal parameters found to be 300 decision trees, 10 features per tree and 4 samples to split branches. Model performance was re-calculated and reported using the average accuracy across 200 internal 5-fold cross-validations (Fig. S1C).

To limit errant predictions, the model-computed probabilities of both correct and incorrect predictions were recorded for each serotype based on the training dataset in cross-validation. For each sample, the serotype with the highest prediction probability was saved and noted as a correct or incorrect classification compared to their labelled serotype. The probability distributions of correct and incorrect classifications were used to fit a generalized linear model with a binomial distribution for each of 17 serotypes that returned incorrect predictions during model training. For cases where the two distributions did not overlap, a minimum probability threshold was determined as (ln(*P*/(1 − *P*)) − *b*_0_)/*b*_1_, where *b*_0_ is the fitted intercept, *b*_1_ the slope and *P*=0.05. For cases where the distributions did overlap, the minimum threshold was calculated using the upper limit of the one-sided 95 % confidence interval of the incorrect classification distribution.

### Feature alignment for closely related serotypes

Reference sequences for *wciZ* (serotype 15B), *wciX* (serotype 18C) and *wciG* (serotype 35B) were obtained from annotated genomes at NCBI (accessions CR931664, CR931673 and KX021817, respectively). blastn [19] was used to obtain the sequence of the corresponding gene for each serotype, and the resulting reading frame was assessed for the presence of a premature stop codon.

### Validation with an external dataset

A collection of NGS data for 2065 UK isolates from Public Health England was used for validation. Reads were *de novo* assembled to genome sequences using SPAdes (v3.14.0, -isolate mode) [20] and serotypes predicted using PfaSTer. Isolates that were previously labelled through latex agglutination [13] to be non-typeable, or serotypes not supported by PfaSTer, were excluded from the calculations. This resulted in validation against 2 026 samples (Table S2). PfaSTer-predicted serotypes were compared to latex agglutination results as well as predictions made by both PneumoCaT and SeroBA – previously reported by Epping *et al*. [13], and predictions made by PneumoKITy as reported by Sheppard *et al*. [15] (Note S1).

## Results

We sought to develop a method for predicting pneumococcal serotypes relying only on minimal data in the form of assembled genome sequences. To this end, we first applied the MinHash (Mash) algorithm, a dimensionality-reduction technique that can effectively compress up to entire genome sequences into a small collection (or *sketch*) of several thousand sub-sequences (k-mers) [17]. As the capsular polysaccharide is encoded at the *cps* operon, we started by performing a Mash Screen [18] comparing 3681 pneumococcal genomes against a k-mer sketch of each serotype’s *cps* locus. The number of matched k-mers to each serotype was then used as features to train a Random Forest classifier. This method predicted the pneumococcal serotype based on the collective voting of hundreds of decision tree estimators, each trained on a bootstrapped set of the 3681 training samples.

After determining the optimal k-mer size (Fig. S1) and hyperparameters, we found the resulting model yielded a median accuracy of 97.8 % in our training data across 200 internal cross-validations. To account for misclassification from low-confidence predictions, we recorded the prediction probabilities returned by the Random Forest model during training and calculated the probability distributions of correct and incorrect serotype calls. We then set probability thresholds based on the 95 % confidence intervals, flagging serotype predictions below these values as low-confidence (Fig. S2).

Following this addition, the major remaining source of misclassifications resulted from closely related serotypes, which could not be distinguished using the Mash Screen results due to a high density of shared k-mers (Fig. S3). In particular, the serotype pairs 15B/C, 18B/C, 24B/F and 35B/D exhibited specificity below 0.87 during cross-validation (Table S3), lower than any other serotypes. While the genetic cause of the 24B and 24F capsular polysaccharides has previously been hypothesized and studied [6, 21], the exact mechanism underlying their differing polysaccharide structures is still unclear. As we could not reliably distinguish serotype 24B from 24F at the time, PfaSTer reported Serogroup 24 when the model predicted either of these serotypes. In contrast, modifications that inactivate genes coding for *O*-acetyltransferases (*wciZ* for 15B/C, *wciX* for 18B/C and *wxiG* for 35B/D) [22–24] impact polysaccharide structure and serotype designations. These modifications can include indels as well as single nucleotide variants (SNVs), leading to frame shifts and/or premature stop codons. Unfortunately, subtle and heterogeneous modifications that inactivate a step in polysaccharide biosynthesis, and therefore polysaccharide structure, were generally not detectable with the Mash Screen technique.

To overcome this challenge in classifying 15B/C, 18B/C and 35B/D isolates, we added a local alignment stage when one of these serotypes was predicted by our model. This step searched the corresponding acetyltransferase for premature termination that would inactivate the protein. By applying this check, we were able to successfully assign each isolate to the correct serotype.

**Fig. 2.**
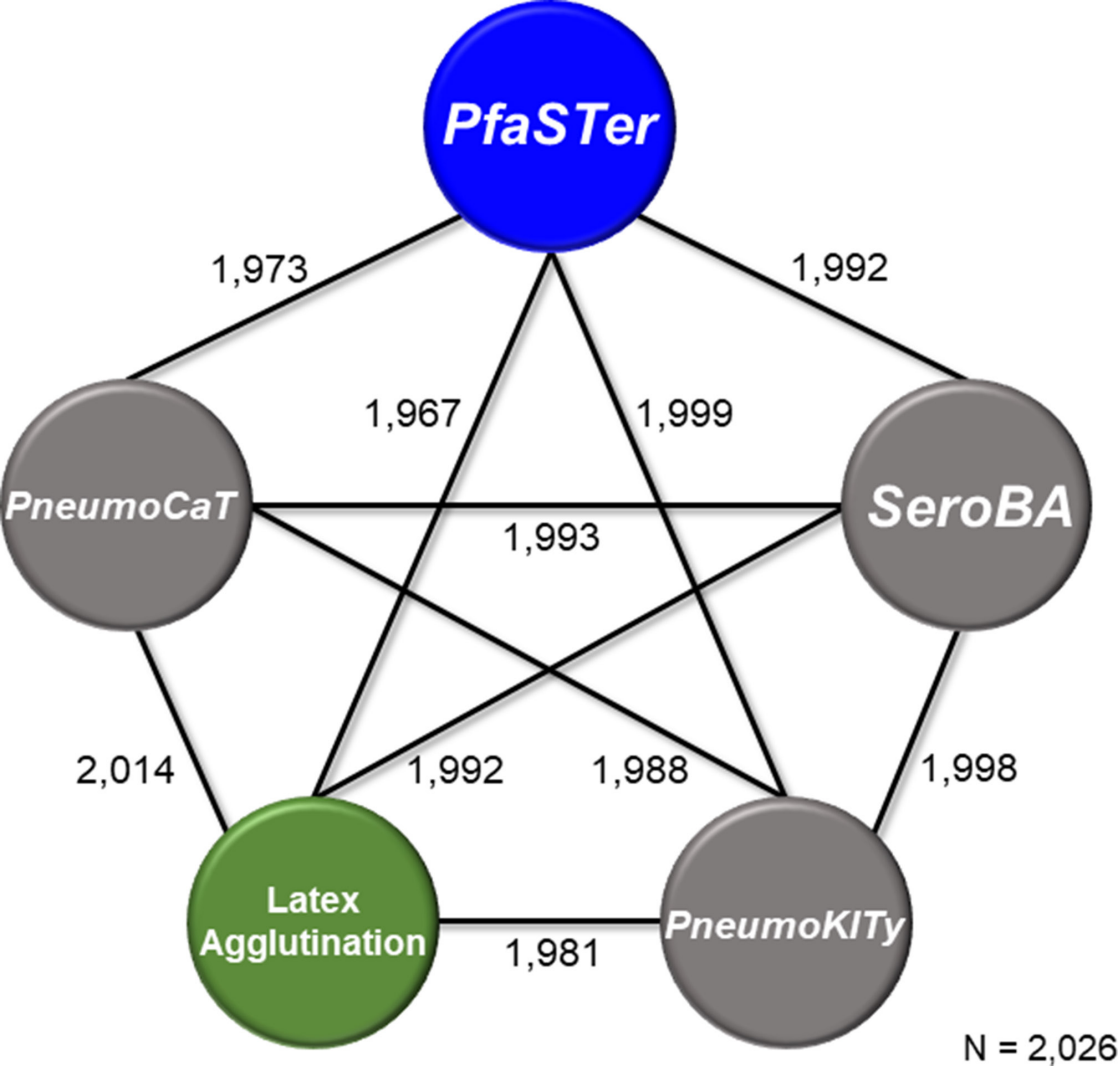
Concordance between serotyping methods for a validated dataset. Number of isolates (out of 2026) returning the same serotype when tested using PfaSTer, latex agglutination and three other *in silico* serotyping tools.

As a final validation, we applied the PfaSTer prediction pipeline to 2026 isolates previously evaluated using the *in silico* serotyping tools PneumoCaT [12] and SeroBA [13], both of which utilize NGS read data as inputs. PfaSTer exhibited high sensitivity for identifying *S. pneumoniae *, returning a serotype in 99.16 % of supported cases (Table S2). Compared to results from latex agglutination, PfaSTer showed 97.09 % concordance in its serotype predictions (Fig. 2, Table S2). This is similar to the ~98 % concordance previously reported by Epping *et al*. using PneumoCat and SeroBA [13]. Furthermore, serotype calling by PfaSTer was in high concordance with the other computational methods, returning the same serotype as PneumoCaT in 97.97 % of cases and SeroBA in 98.47 % of cases (Fig. 2, Table S2). Predictions were also compared to results published for the PneumoKITy serotyping tool [15], for which PfaSTer achieved 98.66 % concordance (Fig. 2, Table S2). PfaSTer also demonstrated an extremely rapid runtime during this benchmarking, with all 2026 samples completed in ~92 min (approximately 2.72 s per genome) on a 36 cpu Amazon EC2 c4 instance running eight parallel processes.

Seventeen isolates, spanning 11 serotypes (Table S4), were not typed by PfaSTer due to prediction probabilities falling below our computed thresholds. For cases where PfaSTer predicted a serotype with high confidence, most disagreement with other typing methods occurred with 15C and 35D designations (Table S2). There were 19 instances where results from latex agglutination, PneumoCaT and SeroBA differed from not only PfaSTer, but also from one another when calling the serotypes 15B or 15C. PfaSTer also identified six isolates as serotype 35D, which were identified as 35B by PneumoCaT and latex agglutination. As an additional validation, we saved the *wciG* alignments for the six 35D predictions, and the *wciZ* alignments for five randomly selected 15C predictions that differed from latex agglutination. We then reviewed the resulting protein sequences for truncation. In all cases, a premature stop was indeed observed in the corresponding *O*-acetyltransferase (Fig. S4). These isolates are, therefore, expected to express 15C or 35D capsular polysaccharide, as predicted by PfaSTer.

Finally, we tested PfaSTer’s specificity for *S. pneumoniae* using 10 *Streptococcus mitis* genomes of diverse collection time, geographical location and multi-locus sequence type (Table S5). Differentiating *S. mitis* and *S. pneumoniae* has been challenging, even with more contemporary molecular typing methods (antigen testing, 16S rRNA gene sequencing or MALDI-TOF MS) [25, 26]. Genome sequence data provide an ultimate resolution to distinguish these two closely related species. PfaSTer was able to differentiate the two, returning negative results (*No Type*) for each of the *S. mitis* genomes analysed (Table S5).

## Discussion

We have developed an efficient tool for rapid *in silico* serotyping of *S. pneumoniae* from assembled genome sequences. This method uses a single-pass k-mer screen and a machine learning model to predict the *S. pneumoniae* serotype without needing to access raw NGS data. While a targeted alignment step is included to resolve a small subset of serotype-specific features (a limitation shared among serotyping pipelines [12, 13]), high-density read data are not needed, in contrast to most other published tools.

A major challenge in developing PfaSTer was establishing confidence in the serotype predictions when constrained to a single genome sequence. While most other *in silico* algorithms designed to assign serotype utilize per-base or per-k-mer coverage to generate statistical confidence, this information is unavailable when working with a single assembled genome. Although the Mash Screen results estimate sequence similarity to each serotype, relative k-mer abundance does not provide any statistical significance on its own. The resulting complications were observed by the authors of the PneumoKITy pipeline, a recently developed alternative for serotype calling from raw NGS data or assembled genomes [15]. Similar to PfaSTer, PneumoKITy utilizes the Mash Screen technique to rapidly identify k-mers matching known *cps* sequences. Serotype prediction is then performed using a pass/fail criterion with a pre-determined threshold based on the authors’ empirical testing: >70 % of k-mers matched for any given serotype. This approach to serotype assignment naturally struggles when the Mash Screen returns a low percentage of k-mer matches, or if >70 % of k-mers are matched for multiple closely related serotypes. In these cases, either no serotype is predicted or multiple potential serotypes are assigned.

PfaSTer addresses this challenge at the machine learning step by leveraging the innate properties of the Random Forest model. As the model consists of an ensemble of decision trees, a prediction probability can be estimated as the proportion of trees agreeing on the serotype [27]. Furthermore, as each decision tree uses a random subset of serotypes as features, PfaSTer leverages presence and absence of k-mers across all 65 included serotypes when computing these probabilities, rather than relying on Mash Screen results against a single serotype at a time. The resulting benefits can be seen when comparing results to PneumoKITy. In the 2026 isolate validation set (Table S2), PfaSTer successfully predicted the serotype of eight isolates where PneumoKITy was unable to identify any serotype due to a shortage of matched k-mers. Additionally, PfaSTer’s machine learning model identified individual serotypes for 339 isolates for which PneumoKITy was restricted to predicting multiple serotypes that all had a high density of k-mer matches. These isolates were spread across serogroups 6, 7, 9, 11, 25, 28 and 33, which did not require PfaSTer’s local alignment module to distinguish.

Of the 2026 isolates used to validate PfaSTer’s performance, a small fraction exhibited consistent discordance with other serotyping methods. This included 17 samples that did not return a serotype due to a low-confidence prediction. Of note, five of these isolates were unable to be typed with other *in silico* pipelines or were reported as a mixture of serotypes (Table S2). This suggests that predictions computed at low probability may be caused in part by low-quality sequencing data.

Discordance was also noted in instances when PfaSTer identified mutations that predict the derived serotypes 15C and 35D. In those samples latex agglutination called the serotype as 15B or 35B, respectively. Notably, each sample with a lack of 15B/15C concordance mapped to mutations in the same region of *wciZ* at a TA-tandem repeat that has been shown to slip and cause indels during replication (Fig. S5) [28, 29]. As repeated frameshifts can convert the serotype between 15B (complete *wciZ* gene and intact *O*-acetyltransferase ORF) and 15C, the serotype can switch over time [12, 28]. Additionally, as antibodies against 15B have been shown to cross-react with 15C polysaccharide [22, 29], mislabelling could occur when typing with antisera. In contrast to 15C, 35D-causing mutations were more widely distributed across *wciG*, causing premature termination at different positions along the protein coding sequence (Fig. S4B).

Although both fast and powerful, serotype assignment using PfaSTer has certain restrictions. As PfaSTer relies on a supervised learning model for prediction, a selection of high-quality genomes must be available for training a generalizable model. While at least 100 pneumococcal serotypes have been recorded [7], certain serotypes are more prevalent than others throughout the world, and published genome sequences are lacking for rare serotypes. For instance, the Sanger Pathogenwatch database (pathogen.watch) currently contains only a single representative genome each for serotypes 10C, 11C, 12A, 33A and 47F, with many other serotypes having fewer than 10 genomes. As a result, PfaSTer prediction is limited to 65 types due to a shortage of available genomes for such rare serotypes. From recent studies, commonly collected serotypes shared across the USA, Europe and Asia include 1, 3, 6A, 6B, 14, 18C, 19F and 23F, with other serotypes identified at a lower frequency [30–34]. These prevalent serotypes are all included in the PCV formulations of PCV13 [35] and PCV20 [9]. To support continual estimation of vaccine coverage, the commonly circulating serotypes in these PCV formulations are all supported by PfaSTer. As the serotype landscape changes over time, and genomes of new isolates are made available, the number of serotypes predicted by PfaSTer may rise.

By relying on an assembled genome, PfaSTer also has reduced functionality for mixed samples compared to some alignment-based serotype tools. For instance, the SeroCall [14] tool can identify both major and minor serotypes in mixed sequencing data by aligning sequencing reads to multiple references. Similarly, PneumoKITy estimates serotype ratios in mixed sequencing data using relative k-mer abundance [15]. While PfaSTer does not support prediction for assembled metagenomes, the presence of each serotype can potentially be inferred from the density of k-mers present in the Mash Screen step [18]. Future developments on PfaSTer could address this feature more directly.

As global monitoring and sequencing of *S. pneumoniae* continues, PfaSTer provides a means to leverage portable, assembled genome sequences for data sharing and serotype tracking. Such surveillance efforts could have an important impact on understanding the spread of *S. pneumoniae* and influence future vaccine design for combatting pneumococcal disease. Finally, this method may have applications suitable for the typing of other microbial species beyond *S. pneumoniae *.

## Supplementary Data

Supplementary material 1

Supplementary material 2

Supplementary material 3
