# Supplementary material for: PfaSTer: a machine learning-powered serotype caller for Streptococcus pneumoniae genomes
Source: Microb Genom. 2023 Jun 6;9(6):mgen001033. doi: 10.1099/mgen.0.001033 (PMC10327508; doi:10.1099/mgen.0.001033)
Supplement: Supplementary material 1 [file mgen-9-1033-s001.pdf]

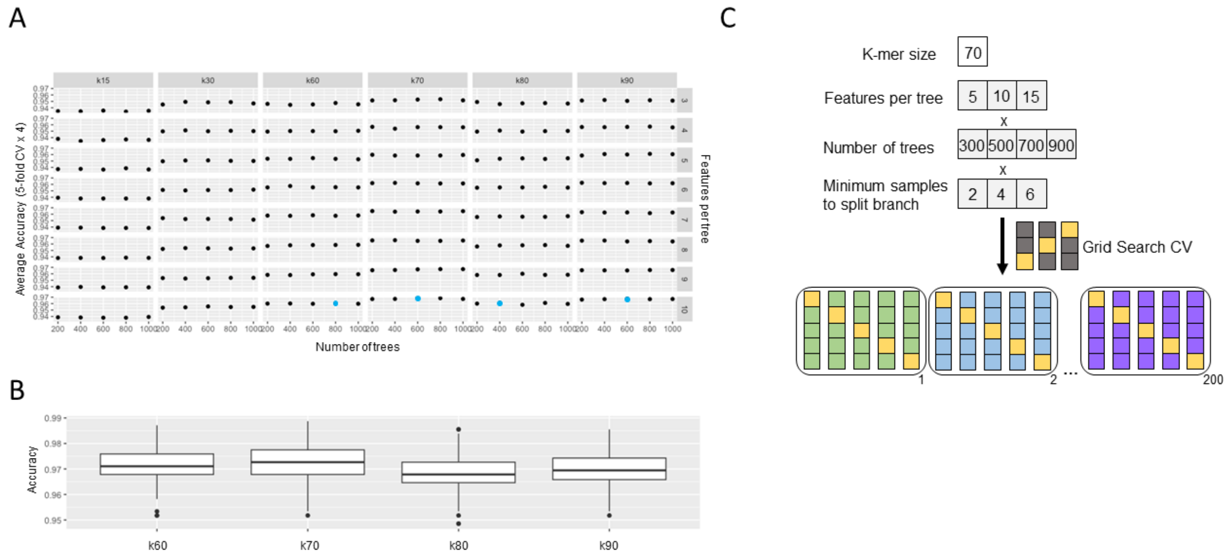

Fig S1. K-mer size selection and model hyperparameter tuning. A) A grid search for features per tree and number of trees in the Random Forest classifier was performed using Mash Screen results from the training dataset at six different k-mer sizes. The best performing set of hyperparameters for k60, k70, k80, and k90 are highlighted in blue. B) Using the best hyperparameter combination for k60, k70, k80, and k90, 200 monte carlo cross-validations were performed for each k-mer size. A k-mer length of 70 was found to result in the highest average accuracy. C) For the finalized model built using sklearn, grid search cross-validation was used for hyperparameter tuning with a k-mer size of 70. Training accuracy was then measured in 200 standard 5-fold cross-validations.

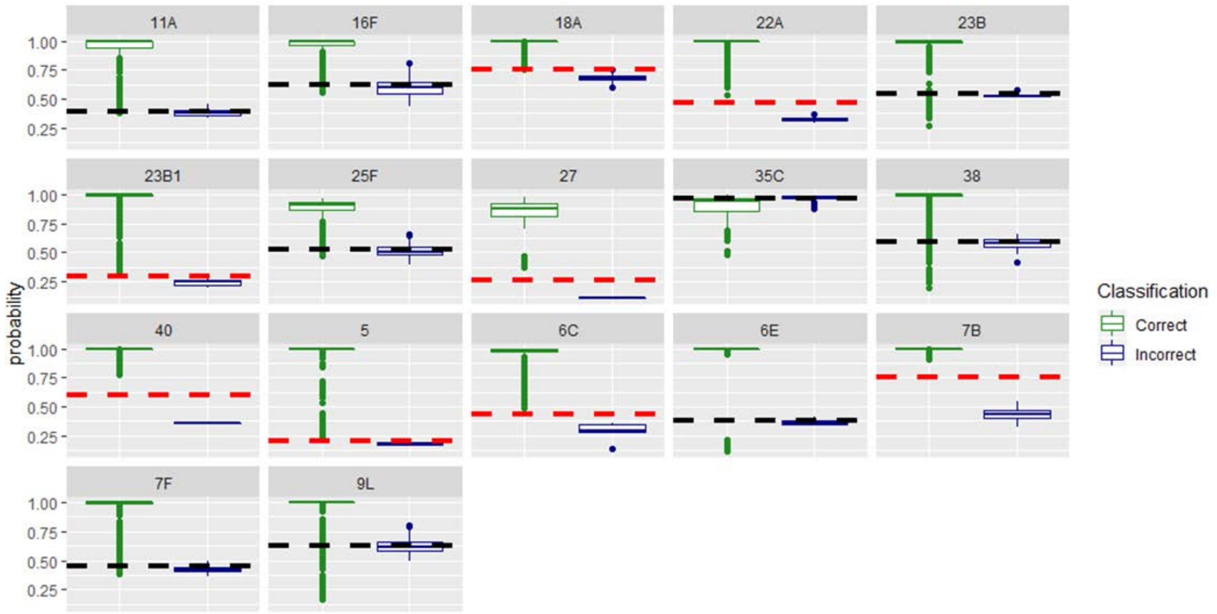

Fig S2: Probability thresholds for 17 serotypes. Model-computed probability distributions for correct and incorrect predictions from cross-validation are shown for 17 serotypes that returned incorrect predictions during model training on 3,681 genomes. Red lines indicate the calculated probability thresholds when distributions do not overlap, and black lines when tails of the distributions do overlap. Predictions made at a probability below these set values are flagged as low-confidence. Calculations for modeling the probability distributions and threshold determination are described in the Materials and Methods. The probability threshold is set at 0.5 for any serotype not included in this figure.

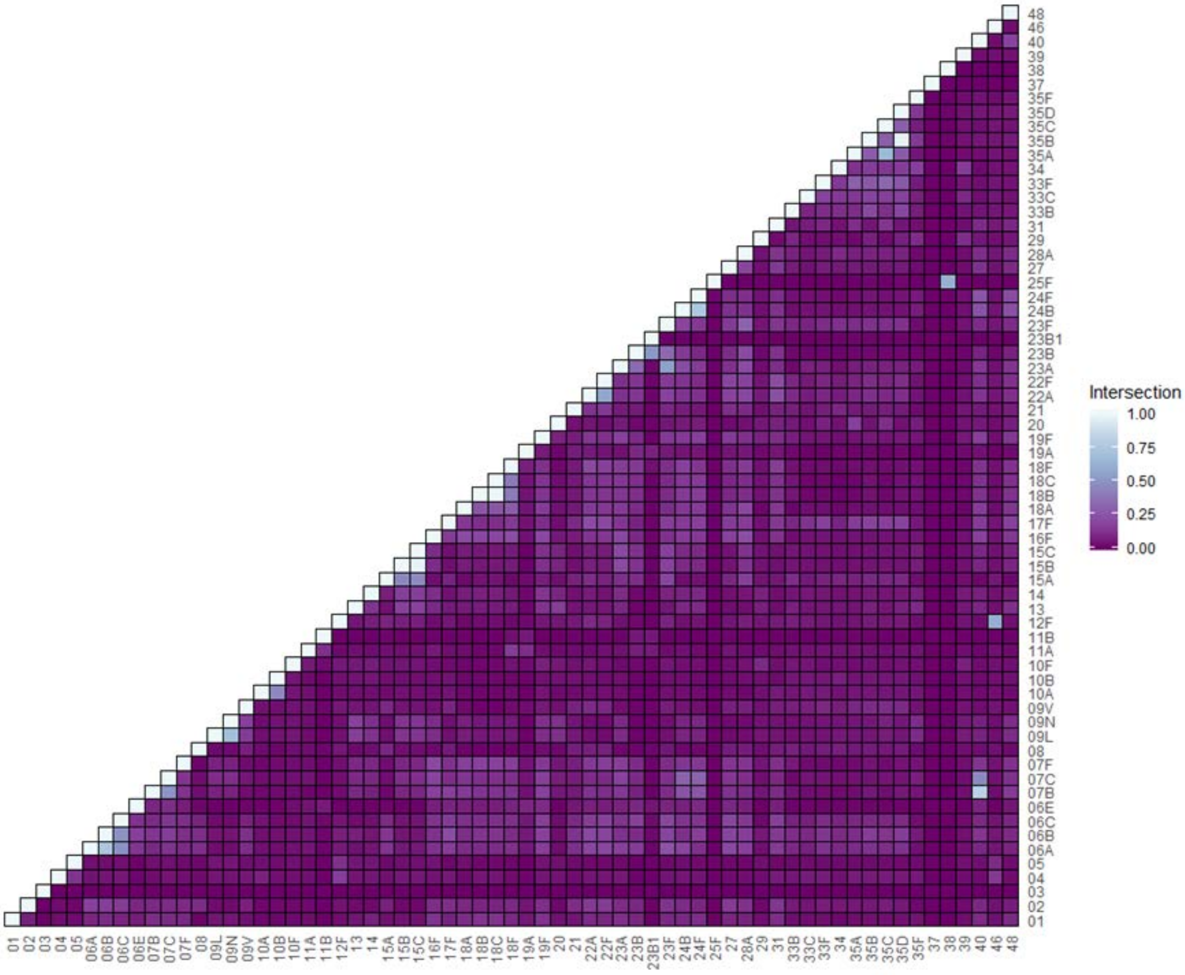

Fig S3: MinHash overlap across serotypes. Fraction of k-mers shared between each pair of 65 serotype MinHash sketches. Proportions correspond to overlap ranging from 0 to 1,000 k-mers.

A

```
             130                      154
WciZ (15B)    PLWYLFAILYIYIYLYFSNKRLLLT
ERR1439297    .............S.FFQ*------
ERR1439407    .............S.FFQ*------
ERR1439409    .............S.FFQ*------
ERR1439342    ...........SLFFQ*--------
ERR1439231    ...........SLFFQ*--------
```

B

```
             94                                               143
WciG (35B)    KRDFSVNPIKKLIGSLVQKGYFFQFWFFGALILIYICLPVLKQFLNSKRS
ERR1440720    ....*---------------------------------------------
ERR1440418    ...................VIS.S.G.LVR.Y.FIYVS.F*---------
ERR1439000    ...........TYRIIGA.RL.LSVLV.WCAYTYLYMS.RFETIS*----
ERR1439452    ..................................................
ERR1439580    ..................................................
ERR1439790    ..................................................

             296                         325
WciG (35B)    FNFIGSYLLFAIFTLSVSFIIVGMLMKIPY
ERR1440720    ------------------------------
ERR1440418    ------------------------------
ERR1439000    ------------------------------
ERR1439452    .........*--------------------
ERR1439580    .........*--------------------
ERR1439790    ..............FCYIYFKC*-------
```

Fig S4: Amino acid sequences of O-acetyltransferases in 15C and 35D isolates predicted by PfaSTer. A) Alignment of a serotype 15B WciZ sequence to five isolates predicted as serotype 15C by PfaSTer. Two variants of WciZ were observed across five isolates, both containing a premature stop prior to residue 150. B) Alignment of a serotype 35B WciG sequence to six isolates predicted to be serotype 35D by PfaSTer. Three sequences terminate prematurely prior to residue 140, and three terminate further downstream prior to residue 320.

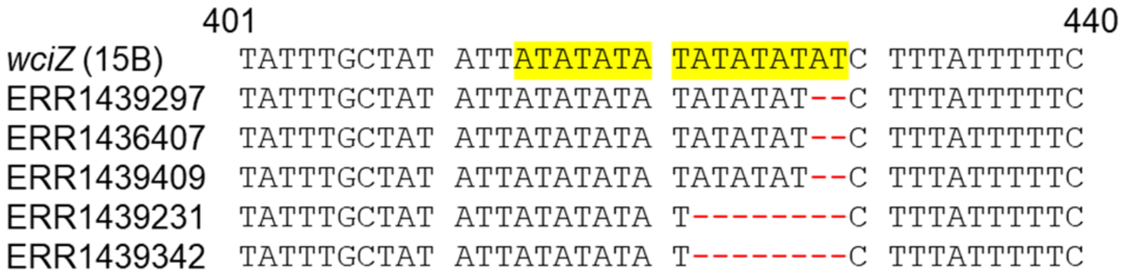

Fig S5: Frameshift mutations at a tandem repeat region of *wciZ* in serotype 15C. Isolates predicted to be serotype 15C by PfaSTer carry multiple nucleotide deletions (red) in an AT-rich tandem repeat (yellow) relative to a 15B reference sequence. Previous work has shown that the resulting frameshift inactivates the WciZ protein and causes formation of the 15C capsular polysaccharide.

Table S1: Isolate names (pathogen.watch) and serotypes for samples used in PfaSTer training.

Table S2: ENA accessions and serotype caller results for isolates used in external validation.

Table S3: Per-class specificity for each serotype during cross validation.

Predicted Serotype	Specificity	Local Alignment Needed
1	1	No
2	1	No
3	1	No
4	1	No
8	1	No
13	1	No
14	1	No
20	1	No
21	1	No
29	1	No
31	1	No
34	1	No
37	1	No
39	1	No
46	1	No
48	1	No
10A	1	No
10B	1	No
10F	1	No
11B	1	No
12F	1	No
15A	1	No
17F	1	No
18F	1	No
19A	1	No
19F	1	No
22F	1	No
23A	1	No
23F	1	No
28A	1	No
33B	1	No
33C	1	No

Predicted Serotype	Specificity	Local Alignment Needed
33F	1	No
35A	1	No
35F	1	No
7C	1	No
9N	1	No
9V	1	No
40	0.9995	No
5	0.9987	No
27	0.9986	No
6C	0.9979	No
23B	0.9976	No
7F	0.9971	No
6E	0.9967	No
23B1	0.9962	No
38	0.9952	No
22A	0.993	No
11A	0.9893	No
18A	0.9849	No
16F	0.9799	No
7B	0.9782	No
9L	0.9778	No
6B	0.9624	No
25F	0.9594	No
35C	0.9511	No
6A	0.9107	No
18C	0.8623	Yes
24F	0.8199	Not applicable
18B	0.8119	Yes
35B	0.7946	Yes
35D	0.7418	Yes
15B	0.5357	Yes
15C	0.5352	Yes
24B	0.0252	Not applicable

Table S4: PfaSTer false negative results per serotype during external validation.

Agglutination serotype	PfaSTer QC fails
3	2
8	1
38	3
10F	1
11A	3
18A	1
18C	2
19F	1
23F	1
28A	1
6A	1

Table S5: Serotype predictions for *S. mitis* genomes.

PubMLST ID	Country	Year	MLST Type	PfaSTer prediction	Prediction Probability
1	United Kingdom	2020	141	NT (No Type)	0.44
27	Gambia	2015	87	NT (No Type)	0.38
155	Denmark	2004	128	NT (No Type)	0.41
172	South Africa	1986	109	NT (No Type)	0.41
174	China	2013	69	NT (No Type)	0.33
188	USA	2015	45	NT (No Type)	0.39
198	USA	2019	8	NT (No Type)	0.41
244	The Gambia	2015	64	NT (No Type)	0.3
296	United Kingdom	2020	53	NT (No Type)	0.36
312	United Kingdom	2020	112	NT (No Type)	0.41

Note S1.

Serotype 23B1 was not included in agglutination assays – instead labeled 23B, but was recognized by both PneumoCaT and SeroBA. In these instances, PfaSTer predictions were considered in concordance when also returning 23B1. Additionally, the published results for SeroBA did not distinguish 6A/6E and 6B/6E at the time, so the subtype 6E was considered in concordance when also predicted by PfaSTer. In cases where latex agglutination or another in-silico method returned multiple serotypes (or an entire serogroup), all the serotypes listed were taken into account during comparison to PfaSTer.
